# The Interrelationship between Ventilatory Inefficiency and Left Ventricular Ejection Fraction in Terms of Cardiovascular Outcomes in Heart Failure Outpatients

**DOI:** 10.3390/diagnostics10070469

**Published:** 2020-07-10

**Authors:** Shyh-Ming Chen, Lin-Yi Wang, Po-Jui Wu, Mei-Yun Liaw, Yung-Lung Chen, An-Ni Chen, Tzu-Hsien Tsai, Chi-Ling Hang, Meng-Chih Lin

**Affiliations:** 1 Section of Cardiology, Department of Internal Medicine, Kaohsiung Chang Gung Memorial Hospital and Chang Gung University College of Medicine, Kaohsiung 83301, Taiwan; sky1021@cgmh.org.tw (P.-J.W.); feymanchen@yahoo.com.tw (Y.-L.C.); garytsai@adm.cgmh.org.tw (T.-H.T.); samuelhang@hotmail.com (C.-L.H.); 2 Department of Physical Medicine and Rehabilitation, Kaohsiung Chang Gung Memorial Hospital and Chang Gung University College of Medicine, Kaohsiung 83301, Taiwan; s801121@cgmh.org.tw (L.-Y.W.); meiynliaw@cgmh.org.tw (M.-Y.L.); 3 Department of Physical Therapy, Kaohsiung Chang Gung Memorial Hospital, Kaohsiung 83301, Taiwan; anni@cgmh.org.tw; 4 Section of Pulmonary and Critical Care Medicine, Department of Internal Medicine and Chang Gung University College of Medicine, Kaohsiung Chang Gung Memorial Hospital, Kaohsiung 83301, Taiwan; mengchih@cgmh.org.tw

**Keywords:** heart failure, mortality, ejection fraction, cardiopulmonary exercise test, ventilatory inefficiency

## Abstract

The relationship between left ventricular ejection fraction (LVEF) and cardiovascular (CV) outcome is documented in patients with low LVEF. Ventilatory inefficiency is an important prognostic predictor. We hypothesized that the presence of ventilatory inefficiency influences the prognostic predictability of LVEF in heart failure (HF) outpatients. In total, 169 HF outpatients underwent the cardiopulmonary exercise test (CPET) and were followed up for a median of 9.25 years. Subjects were divided into five groups of similar size according to baseline LVEF (≤39%, 40–58%, 59–68%, 69–74%, and ≥75%). The primary endpoints were CV mortality and first HF hospitalization. The Cox proportional hazard model was used for simple and multiple regression analyses to evaluate the interrelationship between LVEF and ventilatory inefficiency (ventilatory equivalent for carbon dioxide (VE/VCO2) at anaerobic threshold (AT) >34.3, optimized cut-point). Only LVEF and VE/VCO2 at AT were significant predictors of major CV events. The lower LVEF subgroup (LVEF ≤ 39%) was associated with an increased risk of CV events, relative to the LVEF ≥75% subgroup, except for patients with ventilatory inefficiency (*p* = 0.400). In conclusion, ventilatory inefficiency influenced the prognostic predictability of LVEF in reduced LVEF outpatients. Ventilatory inefficiency can be used as a therapeutic target in HF management.

## 1. Introduction

Heart failure (HF) is a leading cause of cardiovascular (CV) mortality and hospitalization. Preventing hospitalization in HF patients, such as using a multidisciplinary treatment strategy, has become a great priority for clinicians, researchers, and policymakers [1]. In addition to clinical demographic risk factors, left ventricular ejection fraction (LVEF) determined by echocardiography is the most commonly used parameter for the diagnosis and management of stable chronic HF patients [2,3]. The relationship between LVEF and CV outcome is well documented in patients with low LVEF HF [4]. However, LVEF is less useful as a prognostic indicator when it is >45% [5,6]. Thus, reliable assessment of prognosis and risk stratification remain challenges in HF outpatients across the full spectrum of LVEF.

The cardiopulmonary exercise test (CPET) is a useful tool in all stages of HF patient management, from diagnosis to risk assessment [7]. In the past several decades, the peak oxygen uptake (peak VO2/kg) from CPET was considered as the best predictor of 1- to 3-year event-free survival after HF [8]. In some patients, ventilatory inefficiency during exercise may be a superior predictor of prognosis compared to peak VO2/kg [9,10].

Pulmonary abnormalities, such as impaired lung mechanics and abnormal alveolar-capillary gas exchange, may be caused by respiratory comorbidities or HF itself [11]. In stable HF outpatients, whether the relationship between LVEF and CV outcome is affected by ventilatory inefficiency remains unknown. In this study, we hypothesized that the presence of ventilatory inefficiency influences the prognostic predictability of LVEF in stable chronic HF patients.

## 2. Materials and Methods

### 2.1. Subjects

A retrospective cohort of 169 HF outpatients with exercise intolerance took the CPET at a tertiary referral center between May, 2007, and July, 2010. Patients with concurrent signs and symptoms of HF (New York Heart Association functional class II–IV) and evidence of structural heart disease (increased left atrial size or left ventricle hypertrophy) were recruited consecutively. Diagnosis was established by the attending physicians with elevated cardiac biomarker (BNP > 100 pg/mL). Ischemic cardiomyopathy was defined as HF with the presence of severe coronary artery disease or a history of myocardial infarction. Valvular cardiomyopathy was defined as HF caused by primary disease of one of the four heart valves. Dilated cardiomyopathy was defined as dilation and impaired left ventricle contraction, in which primary and secondary causes of heart disease (e.g., coronary artery disease and myocarditis) were excluded. Patients who had a history of HF hospitalization within 6 months or were unable to perform an exercise test were excluded from the study. The patients were followed up at a median of 9.25 years (interquartile range (IQR), 7.48–10.32 years) since the administration of CPET. LVEF was assessed by quantitative echocardiography using the biplane Simpson method. This study was approved by the Institutional Review Board of the Kaohsiung Chang Gung Memorial Hospital (201701459B0, 13th October 2017) and was conducted in accordance with the Helsinki Declaration of 1975 (as revised in 1983). This study was registered at ClinicalTrials.gov (identifier: NCT04141345). Informed consent was obtained prior to CPET administration in all subjects.

### 2.2. CPET Procedures

Patients performed an upright graded bicycle exercise using an individualized protocol. The heart rate was continuously monitored by electrocardiography at rest and during exercise. Blood pressure was measured using an electronic sphygmomanometer (SunTech Medical, Morrisville, NC, USA) every 2 min and as needed. The minute ventilation (VE), oxygen consumption (VO2), and carbon dioxide production (VCO2) were continuously recorded every 1 min using a respiratory mass spectrometer (Vmax Encore, VIASYS, Yorba Linda, CA, USA). Prior to each respiratory gas analysis study, the mass spectrometer was calibrated with a standard gas of known concentration. The peak VO2/kg and the peak respiratory exchange ratio (RER) were defined as the highest 30-s average value obtained during exercise. The anaerobic threshold (AT) was determined using the V-slope method. The VE/VCO2 at AT was calculated as the average VE/VCO2 for 1 min during AT and immediately after AT. If the AT could not be determined, the lowest VE/VCO2 was determined by averaging the three lowest consecutive 0.5-min data points. Since the variability of VE/VCO2 at AT is slightly lower than the variability of the slope of VE versus VCO2 below the ventilatory compensatory point [12,13], this study used VE/VCO2 at AT as a marker of ventilatory efficiency. Spirometric measurements included lung vital capacity, forced vital capacity, forced expiratory volume in 1 s, and maximal voluntary ventilation.

The criteria for discontinuing the test were as follows: request by the subject, threatened arrhythmia, peak RER >1.1, and ≥2.0 mm of horizontal or downslope ST segment depression during progressive exercise. The CPET exams were conducted by a qualified physical therapist under the supervision of a physician.

### 2.3. Outcome Analysis

Defined time-dependent CV outcomes included CV mortality and first HF hospitalization, which were the primary endpoints of the analysis. Study subjects were followed until the end of 2018. HF hospitalization was defined as an unplanned hospitalization due to new or worsening HF requiring the use of intravenous diuretics, inotropes, or vasodilators.

### 2.4. Statistical Analyses

Subjects were divided into five groups of similar size according to baseline LVEF (≤39%, 40–58%, 59–68%, 69–74%, and ≥75%) by each 20-percentile sample size to evaluate the relationship between LVEF and CV outcomes. Comparisons between LVEF groups were analyzed using Pearson’s chi-square test or Fisher’s exact test for categorical variables. Continuous variables were expressed as median (IQR). Comparisons between LVEF groups were analyzed using the Kruskal–Wallis test and multiple comparisons for continuous variables. The Kolmogorov–Smirnov test was used to test for normality. For the univariate and multivariable analyses, the hazard ratio and 95% confidence interval were computed using the Cox proportional hazard model. The variables in which *p* value was <0.1 by univariate analysis were included on multivariate analysis and stepwise method. The primary endpoint was defined as CV mortality or the first HF hospitalization. The comparative results of primary endpoints between patients with LVEF ≥50% (HFpEF–i.e., HF with preserved ejection fraction (EF)) and those with LVEF <50% (non-HFpEF–i.e., mid-range (LVEF 40–49%) and reduced EF (LVEF < 40%)) were analyzed. The various CPET parameters were evaluated as predictors of primary endpoints by performing time-dependent receiver operating characteristic curve (ROC) analyses. Optimized threshold values for VE/VCO2 at AT were identified via ROC analysis and the Youden index. The Cox proportional hazard model was used for simple and multiple regression analyses to evaluate the interrelationship between LVEF and ventilatory inefficiency (defined as VE/VCO2 at AT >34.3, optimized cutoff point). The interaction term “ventilatory inefficiency multiplied by LVEF category” was introduced to the previous model. Kaplan–Meier survival curves were constructed for five groups of patients according to baseline LVEF. Data were analyzed using R v3.6.1 software using “time ROC” and “survival” package and SPSS 22.0 (SPSS Inc., Chicago, IL, USA). In all analyses, a *p* value of less than 0.05 was considered statistically significant.

## 3. Results

### 3.1. Baseline Clinical and Pharmacological Characteristics by LVEF

The mean LVEF in our HF outpatients was 64.0 ± 18.6%. The baseline clinical demographic and pharmacological characteristics according to LVEF are shown in Table 1. Patients with higher EF were more often female and were more likely to have a history of hypertension. Patients with lower EF were more likely to have a history of smoking, ischemic cardiomyopathy, and/or received percutaneous coronary intervention (PCI). Patients who suffered from dilated cardiomyopathy had lower EF. The incidence of diabetes, valvular heart disease, and ischemic stroke did not differ across these LVEF subgroups. The distribution of age also did not differ significantly across the LVEF subgroups. The proportion of patients who received beta-blockers, angiotensin-converting enzyme inhibitors (ACEIs), angiotensin-receptor blockers (ARB), loop diuretics, and mineralocorticoid receptor antagonists (MRAs) increased in the lower EF patients. In contrast, the proportion of patients who received dihydropyridine (DHP) calcium (Ca^+^) channel blockers increased in the higher EF patients. The CPET parameters including peak VO2/kg, AT, ΔVO2/ΔWR and VE/VCO2 at AT had a significant difference across the spectrum of LVEF (Table 1).

### 3.2. Outcomes by LVEF

Within a median follow-up period of 9.25 years (IQR, 7.48–10.32 years), 49 patients achieved our primary endpoints. The relationship between LVEF and the primary endpoints, including CV mortality, is shown in Table 2A. The risk of primary endpoints and CV mortality was increased in the lower LVEF subgroups (*p* = 0.002 and 0.001, respectively). HFpEF patients had better CV outcomes compared with non-HFpEF patients (primary endpoints and CV mortality: *p* = <0.0001 and 0.001, respectively). There were similar CV outcomes of HFpEF who had ventilatory inefficiency and those with non-HFpEF (primary endpoints and CV mortality: *p* = 0.792 and 0.358, respectively) (Table 2B).

### 3.3. Univariate and Multivariate Analysis of Predictors of Major Cardiovascular Events

**Figure 1:**
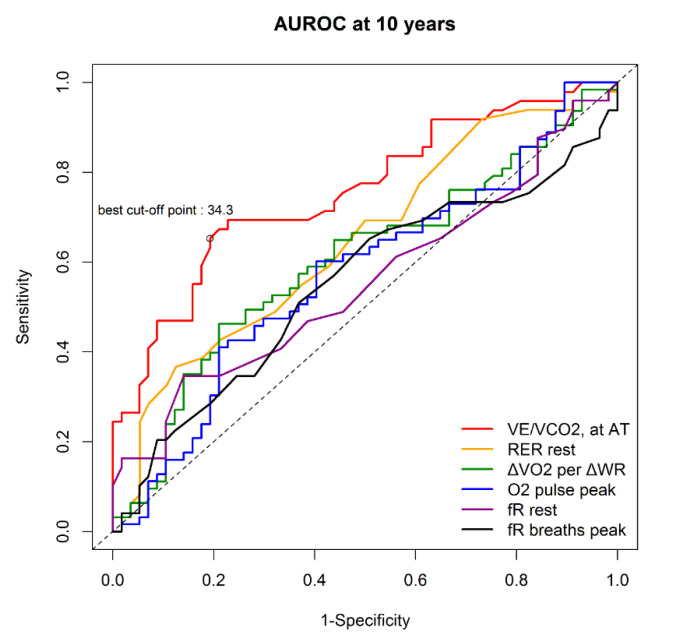
In ROC analyses of different CPET parameters, the only significant predictor of heart failure hospitalization was the VE/VCO2 at AT. Best cut-off point: 34.3, AUROC: 0.756. VE/VCO2 at AT: ventilatory equivalent for carbon dioxide at anaerobic threshold, RER: respiratory exchange ratio; ΔVO2/ΔWR: the ratio of increase in oxygen uptake to increase in work rate; fR rest: resting breathing rate; fR breath peak: peak exercise breathing rate; AUROC: area under receiver operating characteristic curve; CPET: cardiopulmonary exercise test.

Table 3 shows that, according to the univariate Cox regression analysis, the significant predictors of major CV events included comorbidities with lung disease, diabetes, LVEF, or dilated cardiomyopathy, a history of smoking, and treatments with beta-blockers, loop diuretics, or MRAs. The CPET parameters, including VE/VCO2 at AT, ΔVO2/ΔWR, peak O2 pulse, peak VO2, peak VO2/kg, peak work, and AT, were significant predictors for major CV events, based on the univariate analysis. In the multivariate Cox regression analyses and stepwise method, which included those variables in which *p* was <0.1 by univariate analysis, only LVEF and VE/VCO2 at AT were found to be significant predictors of major CV events in our cohort study (Table 3). The optimized threshold value of VE/VCO2 at AT was identified by ROC analysis. For predicting primary endpoints in all patients, the best cutoff point for VE/VCO2 at AT was 34.3 (64.3 sensitivity and 78.0% specificity, Youden index = 0.42) (Figure 1).

### 3.4. Adjust Hazard Ratio Associated with LVEF for Major Cardiovascular Events by Baseline LVEF Category Relative to LVEF ≥ 75

**Figure 2:**
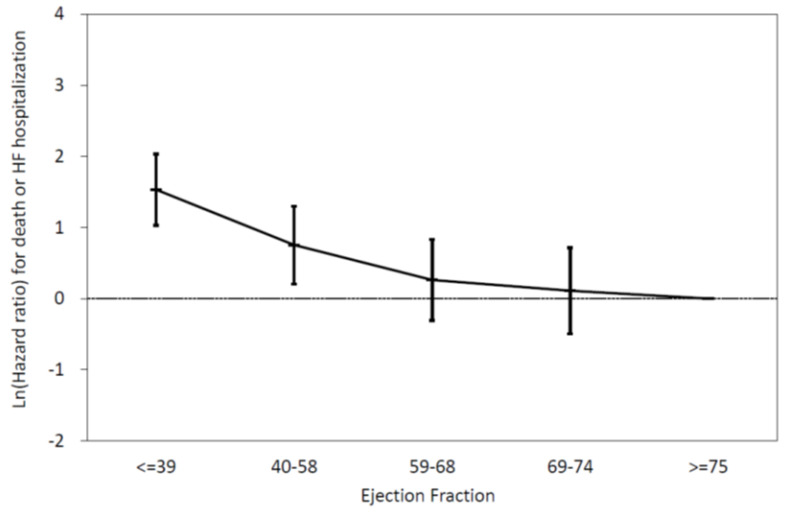
The relationship between LVEF and CV outcomes in all patients. This relationship was not linear. The lower LVEF subgroup (LVEF ≤ 39%) was associated with a significantly increased risk of CV mortality or HF hospitalization relative to the LVEF ≥75% subgroup. (*p* = 0.002). LVEF: left ventricular ejection fraction; CV: cardiovascular; HF: heart failure.

**Figure 3:**
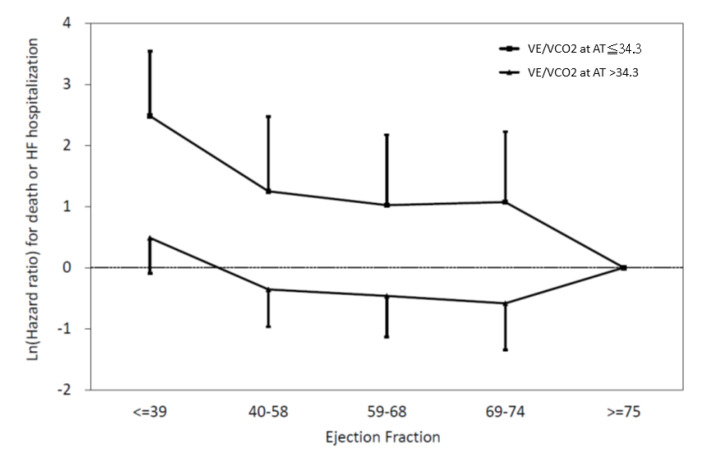
The relationship between LVEF and CV outcomes in patients with ventilatory inefficiency (VE/VCO2 at AT >34.3) and in patients without ventilatory inefficiency (VE/VCO2 at AT ≤34.3). The lower LVEF subgroup (LVEF ≤ 39%) was associated with a significantly increased risk of CV mortality or HF hospitalization relative to the LVEF ≥75% subgroup among patients without ventilatory inefficiency (*p* = 0.019). There was no prognostic predictability relative to low EF (LVEF ≤ 39%) among patients with ventilatory inefficiency (*p* = 0.400). LVEF: left ventricular ejection fraction, CV: cardiovascular, VE/VCO2 at AT: ventilatory equivalent for carbon dioxide at anaerobic threshold, HF: heart failure.

**Figure 4:**
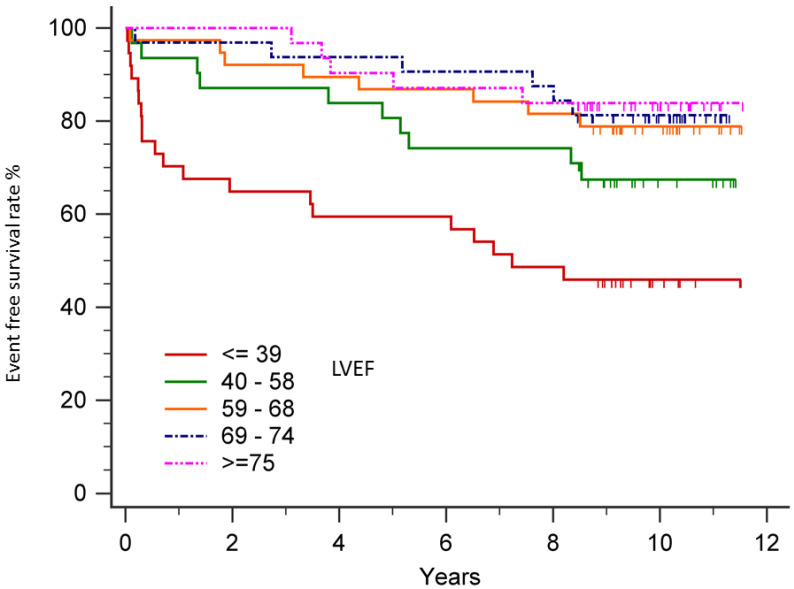
The Kaplan–Meier analysis of five groups of patients with different LVEF. Only patients with LVEF ≤39 had significant survival difference when compared with other groups. LVEF: left ventricular ejection fraction.

As presented in Figure 2, the relationship between LVEF and major CV events was not linear. We defined ventilatory inefficiency as VE/VCO2 at AT >34.3. To characterize the relationship between LVEF and the risk of CV mortality or HF hospitalization among patients with ventilatory inefficiency, subjects were divided into five subgroups according to baseline LVEF. Figure 3 shows the relationship between LVEF and major CV events in patients with ventilatory inefficiency (VE/VCO2 at AT >34.3) and in patients without ventilatory inefficiency (VE/VCO2 at AT ≤34.3). After multivariable adjustment, the Cox proportional hazard model showed that the lower LVEF subgroup (LVEF ≤ 39%) was associated with a significantly increased risk of CV mortality or HF hospitalization relative to the LVEF ≥75% subgroup among patients without ventilatory inefficiency (VE/VCO2 at AT ≤34.3) (*p* = 0.019) and among all patients (*p* = 0.002) (Table 4). Conversely, there was no prognostic predictability relative to low EF (LVEF ≤ 39%) among patients with ventilatory inefficiency (VE/VCO2 at AT >34.3) (*p* = 0.400). However, the interaction effect between LVEF and ventilatory inefficiency in predicting CV major events was not significant (*p* = 0.579). Figure 4 showed the results of Kaplan–Meier analysis of five groups of patients with different LVEF. Among them, only the LVEF ≤39% group showed a significant survival difference (*p* = 0.047 vs. LVEF 40–58%, *p* = 0.002 vs. LVEF 59–68%, *p* = 0.001 vs. LVEF 69–74%, and *p* = 0.001 vs. LVEF ≥75%).

## 4. Discussion

In chronic HF outpatients followed for a median of 9.25 years, LVEF and VE/VCO2 at AT were both found to be significant independent predictors of increased risk of CV mortality or HF hospitalization. LVEF was a poor predictor in patients with ventilatory inefficiency and in those with LVEF >40%. Although our study showed that the interaction effect between LVEF and VE/VCO2 at AT was not significant, the prognostic predictability of LVEF was decreased in the HF with reduced LVEF (HFrEF, LVEF ≤39%) population in the ventilatory inefficiency group. As demonstrated in the CHARM Program [5], the relationship between LVEF and CV outcomes was not linear. We also demonstrated a similar finding in chronic HF outpatients. This relationship was further diminished in the ventilatory inefficiency group. This phenomenon revealed that HFpEF patients who had ventilatory inefficiency had similar CV outcomes as that of their HFrEF counterparts.

This study showed that the ventilation efficiency variable, in addition to LVEF, was a significant prognostic predictor in HF outpatients. Ventilatory inefficiency reflects the adverse effects of HF on lung mechanics and diffusion capacity [14], as HF also augments ventilatory drive and increases hemodynamic demand associated with breathing work [15]. Ergoreceptors stimulate ventilation and activate sympathetic hormones in response to work. The ergoreflex in the muscle also affects ventilatory effort. In response to carbon dioxide and pulmonary J receptors (which likely respond to congestion and alveolar stiffness), central and pulmonary chemoreceptors contribute to the ergoreflex and result in excess ventilation [16]. In HF patients, a high ventilatory drive can reduce the partial pressure of CO_2_ (PaCO2) [17]. Consequently, a reduced PaCO2 and increased fractional dead space cause abnormally high VE/VCO2 at AT, i.e., ventilatory inefficiency [18,19].

The mechanism of ventilatory inefficiency influences the outcomes of HF patients differently between the HFrEF and HFpEF patients. A study analyzed the ventilatory inefficiency between 24 HFrEF patients and 33 HFpEF patients [20]. It demonstrated the loss of cardiac output augmentation related to ventilatory inefficiency regardless of LVEF; however, lung congestion parameters (echocardiographic parameter: e′ and E/e′) correlated with ventilatory inefficiency only in HFpEF. In another study, ventilatory inefficiency appeared to be influenced by mechanisms regulating PaCO2 in HFrEF. In contrast, dead space to tidal volume ratio (VD/VT) played a more important role in developing ventilatory inefficiency in HFpEF [21]. HFpEF and HFrEF may be two distinct entities in terms of ventilatory response to exercise; this study provides evidence that ventilatory inefficiency plays a critical role in HFpEF.

CPET-based measurements of ventilatory inefficiency provide unique physiologic information clinically relevant to contemporary treatment for HF. Several therapeutic interventions for HF affect ventilatory abnormalities both at rest and during exercise. For example, ACEI improves pulmonary diffusion, removes interstitial fluid, and improves pulmonary hemodynamic status [22]. Carvedilol, but not bisoprolol, improves ventilatory efficiency during exercise (reduction of VE/VCO2 slope and increase in maximum end-tidal CO_2_ pressure) [23]. Carvedilol may have direct effects on respiratory chemoreceptor activity based on the CARNEBI (CARvedilol vs. NEbivolol vs. BIsoprolol in moderate heart failure) trial [24]. Carvedilol and bisoprolol are both beta-blockers in this study. CPET can be served as a practical guide for the best selection of different beta-blockers. As ventilatory inefficiency is a significant prognostic predictor across the spectrum of LVEF, we should consider ventilatory abnormalities during exercise as therapeutic targets and treat them accordingly. Therapeutic interventions, such as rehabilitation training (isolated quadriceps training) [25], device-guided paced breathing [26], yoga mantras [27], and reduction of afferent stimuli from ergopulmonary and cardiopulmonary receptors [28,29], might all alleviate ventilatory inefficiency. The use of CPET-derived variables to guide therapy and improve outcomes deserves further investigation.

LVEF has proven largely inadequate in correlating HF patients’ mortality in heart transplant candidate [30]. However, LVEF is still a good predictor of incident HF in outpatient setting. In the CARE trial, LVEF was the significant predictor of HF attack in 3860 long-term survivors of myocardial infarction [31]. In chronic stable condition, LVEF is a prognostic indicator, as shown in our study. However, this discriminatory effect of LVEF in predicting morbidity and mortality was limited in HFpEF and patients with ventilatory inefficiency.

This study has some limitations. First, the sample size was relatively small compared to those in other epidemiological studies. However, our study had a longer follow-up period than those of previous works. Second, patients were only recruited from outpatient clinics, which may have caused selection bias. The findings of this study may need further validation in other populations of patients with HF. Third, this study did not analyze other CPET variables that have been used to predict HF outcomes, e.g., oscillatory ventilation, end-tidal CO_2_ pressure, VO2 kinetics during exercise, oxygen uptake efficiency slope, and heart rate recovery. Therefore, whether the predictive accuracy of these variables can be increased by combining them with VE/VCO2 at AT requires further investigation. The subgroup of HF patients who had improved LVEF had a more favorable prognosis compared with patients whose LVEF had not changed [32]. However, this study focused on clinically assessed LVEF at baseline, which is the actual measurement used to guide patient care and its relationship with outcomes. The change of LVEF was not used as a variable in our analysis.

## 5. Conclusions

Ventilatory inefficiency influenced the prognostic predictability of LVEF in HFrEF patients when compared to patients with LVEF ≥75%. The CPET-derived variable (VE/VCO2 at AT) can be used as a therapeutic target in HF management. However, the interaction effect between LVEF and ventilatory inefficiency in predicting CV outcomes was not significant.

## Figures and Tables

**Table 1:** Baseline clinical and pharmacological characteristics by LVEF.

Variables	All Patients (*n* = 169)	LVEF ≤39% (37)	LVEF 40–58% (31)	LVEF 59–68% (38)	LVEF 69–74% (32)	LVEF ≥75% (31)	*p* Value
Age	55.7 ± 13.5	50.9 ± 14.7	59.6 ± 12.3	54.3 ± 12.8	57.1 ± 14.6	57.7 ± 11.7	0.097
Male	121 (71.6%)	34 (91.9%)	23 (74.2%)	27 (71.1%)	17 (53.1%)	20 (64.5%)	0.008
Lung disease Both (%)	79 (46.7%)	20 (54.1%)	17 (54.8%)	15 (39.5%)	15 (46.9%)	12 (38.7%)	0.522
Obstructive lung (%)	13 (7.7%)	4 (10.8%)	4 (12.9%)	1 (2.6%)	3 (9.4%)	1 (3.2%)	0.396
Restrictive lung (%)	66 (39.1%)	16 (43.2%)	13 (41.9%)	14 (36.8%)	12 (37.5%)	11 (35.5%)	0.956
Hypertension (%)	99 (58.6%)	13 (35.1%)	23 (74.2%)	23 (60.5%)	19 (65.5%)	21 (72.4%)	0.006
Diabetes (%)	37 (22.7%)	9 (24.3%)	10 (32.3%)	10 (26.3%)	4 (13.8%)	4 (14.3%)	0.355
Smoking (%)	39 (23.5%)	16 (43.2%)	8 (25.8%)	7 (18.4)	5 (16.1%)	3 (10.3%)	0.015
Ischemic stroke (%)	9 (5.6%)	0 (0%)	1(3.2%)	2 (5.3%)	2 (6.9%)	4 (14.3%)	0.158
Ischemic CM (%)	33 (19.5%)	15 (40.5%)	10 (32.3%)	2 (5.3%)	4 (12.5%)	2 (6.5%)	<0.0001
Valvular CM (%)	22 (13.0%)	3 (8.1%)	6 (19.4%)	4 (10.5%)	3 (9.4%)	6 (19.4%)	0.497
Dilated CM (%)	24 (14.2%)	15 (40.5%)	7 (22.6%)	2 (5.3%)	0 (0%)	0 (0%)	<0.0001
Prior PCI (%)	29 (17.2%)	13 (35.1%)	9 (29.0%)	2 (5.3%)	4 (12.5%)	1 (3.2%)	0.001
Medication							
Beta-blocker (%)	97 (58.4%)	30 (81.1%)	25 (80.6%)	19 (50.0%)	12 (38.7%)	11 (37.9%)	<0.0001
ACEI/ARB (%)	114 (67.5%)	32 (86.5%)	28 (90.3%)	21 (55.3%)	15 (46.9%)	18 (58.1%)	<0.0001
DHP Ca^+^ channel blocker (%)	36 (21.7%)	1 (2.7%)	10 (32.3%)	5 (13.2%)	11 (35.5%)	9 (31.0%)	0.002
Loop diuretic (%)	43 (25.9%)	22 (59.5%)	13 (41.9%)	3 (7.9%)	4 (12.9%)	1 (3.4%)	<0.0001
MRA (%)	21 (12.4%)	13 (35.1%)	5 (16.1%)	2 (5.3%)	1 (3.2%)	0 (0%)	<0.0001
Statin (%)	53 (31.9%)	13 (35.1%)	9 (29.0%)	12 (31.6%)	10 (32.3%)	9 (31.0%)	0.989
Parameters of CPET							
Peak O2 pulse (mL/beat)	11.9 (9.64–14.89)	11.04 (9.18–15.99)	10.97 (7.78–13.76)	12.16 (9.93–14.92)	12.11 (9.42–15.1)	12.12 (10.11–14.90)	0.303
Peak VO2/kg (mL/kg/min)	22.9 (18.2–28.4)	20.0 (15.9–26.0)	21.3 (16.8–25.1)	25.1 (19.1–29.7)	23.4 (19.5–29.0)	25.5 (19.4–31.9)	0.045
Peak VE (L/min)	54.0 (43.0–65.0)	60.0 (44.5–71.0)	52.0 (37.0–63.0)	59.0 (45.8–68.8)	49.0 (41.0–60.5)	49.0 (43.0–65.0)	0.159
AT (% of VO2 max)	54.9 (45.8–66.2)	50.0 (41.2–60.7)	51.0 (45.7–57.8)	58.2 (49.2–66.4)	56.4 (44.6–73.5)	61.7 (52.2–74.2)	0.007
VE/VCO2 at AT	32.3 (29.2–35.8)	33.4 (29.9–38.1)	34.8 (29.8–37.9)	31.7 (28.8–35.8)	32.0 (28.9–34.1)	30.9 (27.7–33.1)	0.036
Peak RER	1.04 (0.98–1.09)	1.05 (1.02–1.12)	1.02 (0.97–1.09)	1.05 (1.0–1.12)	1.03 (0.96–1.07)	1.04 (0.95–1.07)	0.118
ΔVO2/ΔWR (mL/min/W)	11.6 (9.9–14.3)	10.4 (8.1–12.6)	11.2 (10.2–13.2)	11.8 (9.9–14.4)	11.4 (10.1–14.3)	14.0 (10.8–16.0)	0.015
Peak VO2 (L/min)	1600 (1233–2074)	1528 (1101–2217)	1461 (980–1676)	1668 (1352–2114)	1609 (1245–1982)	1706 (1339–2117)	0.152
Peak Work (Watts)	119.0 (77.5–161.5)	135.0 (69.0–193.5)	96.0 (74.0–125.0)	125.5 (88.5–162.3)	115.5 (79.8–158.5)	123.0 (69.0–158.0)	0.353
Breathing Reserve (L)	28.9 (15.1–42.0)	34.0 (12.8–44.2)	26.2 (10.6–40.0)	30.9 (22.0–42.9)	20.2 (8.5–35.6)	33.2 (18.2–41.6)	0.221

LVEF: left ventricle ejection fraction; CM: cardiomyopathy; PCI: percutaneous coronary intervention; ACEI: angiotensin-converting enzyme inhibitor; ARB: angiotensin receptor blocker; DHP: dihydropyridine; MRA: mineralocorticoid receptor antagonist; CPET: cardiopulmonary exercise test; VO2/kg: oxygen consumption per kilogram; VE: minute ventilation; AT: anaerobic threshold; VE/VCO2 at AT: ventilatory equivalent for carbon dioxide at anaerobic threshold; RER: respiratory exchange ratio; ΔVO2/ΔWR: the ratio of increase in oxygen uptake to increase in work rate.

**Table 2:** (**A**) Outcomes by LVEF (5 groups); (**B**) outcomes between HFpEF and non-HFpEF without or with ventilatory inefficiency.

(**A**)								
**Variables**	**All Patients (*n* = 169)**		**LVEF** **≤39%**	**LVEF** **40–58%**	**LVEF** **59–68%**	**LVEF** **69–74%**	**LVEF** **≥75%**	***p* Value**
Primary endpoints	49 (29%)		20 (54.1%)	10 (32.3%)	8 (21.1%)	6 (18.8%)	5 (16.1%)	0.002
Cardiovascular mortality	18 (10.7%)		10 (27.0%)	5 (16.1%)	2 (5.3%)	0 (0%)	1 (3.2%)	0.001
(**B**)								
**Variables**	**Non-HFpEF**	**HFpEF**	***p* Value**	**Non-HFpEF** **with Ventilatory Inefficiency**		**HFpEF with Ventilatory Inefficiency**		***p*** **Value**
Primary endpoints	27 (48.2%)	22 (19.5%)	<0.0001	17 (58.6%)		15 (51.7%)		0.792
Cardiovascular mortality	12 (21.4%)	6 (5.3%)	0.001	9 (31.0%)		5 (17.2%)		0.358

LVEF: left ventricular ejection fraction; HFpEF: heart failure with preserve ejection fraction (LVEF ≥ 50%); non-HFpEF: heart failure with LVEF <50% (i.e., mid-range (LVEF 40–49%) and reduced ejection fraction (LVEF < 40%); ventilatory inefficiency: VE/VCO2 at AT (ventilatory equivalent for carbon dioxide at anaerobic threshold) >34.3.

**Table 3:** Univariate and multivariate analysis of predictors of major cardiovascular events.

Independent Variable		Univariate Analysis			Multivariate Analysis		
		HR	(95% CI)	*p* Value	HR	(95% CI)	*p* Value
Age at CPET		1.0	(0.99–1.02)	0.966			
Male		1.66	(0.83–3.33)	0.152			
Lung Disease							
	Obstructive	1.45	(0.57–3.65)	0.433			
	Restrictive	1.73	(0.99–3.02)	0.057			
	Both	1.92	(1.09–3.40)	0.025			
Ischemic stroke		1.56	(0.56–4.44)	0.392			
Myocardial infarction		1.31	(0.66–2.63)	0.442			
Hypertension		0.66	(0.37–1.15)	0.139			
Prior PCI		1.74	(0.91–3.34)	0.096			
Diabetes		2.06	(1.14–3.71)	0.016			
Smoking		1.97	(1.10–3.56)	0.024			
LVEF		0.97	(0.96–0.98)	<0.001	0.98	(0.96–0.99)	0.002
Ischemic cardiomyopathy		1.65	(0.88–3.11)	0.122			
Dilated cardiomyopathy		2.03	(1.04–3.98)	0.039			
Valvular cardiomyopathy		1.37	(0.64–2.92)	0.416			
Beta-blocker		2.24	(1.19–4.22)	0.013			
ACEI/ARB		1.88	(0.96–3.69)	0.064			
DHP Ca^+^ channel blocker		0.88	(0.44–1.76)	0.718			
Loop diuretic		3.39	(1.93–5.96)	<0.001			
MRA		4.10	(2.17–7.77)	<0.001			
Statin		1.57	(0.89–2.78)	0.121			
VE/VCO2 at AT		1.19	(1.14–1.25)	<0.001	1.17	(1.12–1.23)	<0.001
ΔVO2/ΔWR (mL/min/W)		1.04	(1.01–1.07)	0.008			
Peak O2 pulse (mL/beat)		0.90	(0.83–0.97)	0.009			
Peak VO2 (L/min)		1.0	(0.99–1.0)	0.001			
Peak RER		0.27	(0.01–5.60)	0.395			
Breathing reserve (mL)		1.00	(0.99–1.01)	0.934			
Peak VE (L/mins)		1.0	(0.98–1.01)	0.731			
Peak VO2/kg (mL/kg/mins)		0.90	(0.85–0.95)	<0.001			
Peak work (Watts)		0.99	(0.99–1.0)	0.009			
Anaerobic threshold		0.95	(0.93–0.97)	<0.001			

Method = forward stepwise selection. HR: hazard ratio; CI: confidence interval; CPET: cardiac pulmonary exercise test; PCI: percutaneous coronary intervention; ACEI: angiotensin-converting enzyme inhibitor; ARB: angiotensin receptor blocker; DHP: dihydropyridine; MRA: mineralocorticoid receptor antagonist; VE/VCO_2_ at AT: ventilatory equivalent for carbon dioxide at anaerobic threshold; ΔVO2/ΔWR: the ratio of increase in oxygen uptake to increase in work rate; peak VO2: peak oxygen consumption; RER: respiratory exchange ratio; VE: minute ventilation; VO2/kg: oxygen consumption per kilogram; AT: anaerobic threshold.

**Table 4:** Adjust hazard ratio associated with LVEF for major cardiovascular events by baseline LVEF category relative to LVEF ≥75.

LVEF Group	VE/VCO2 at AT ≤34.3	*p* Value	VE/VCO2 at AT >34.3	*p* Value	All	*p* Value
	HR (95% CI)		HR (95% CI)		HR (95% CI)	
≤39	12.00 (1.50–96.01)	0.019	1.63 (0.52–5.08)	0.400	4.63 (1.74–12.35)	0.002
40–58	3.49 (0.32–38.48)	0.308	0.70 (0.21–2.33)	0.561	2.12 (0.73–6.22)	0.169
59–68	2.78 (0.29–26.74)	0.376	0.63 (0.17–2.35)	0.492	1.30 (0.42–3.97)	0.647
69–74	2.92 (0.30–28.11)	0.353	0.56 (0.12–2.50)	0.445	1.12 (0.34–3.66)	0.854
≥75	1		1		1	

Interaction term: *p* value = 0.579. LVEF: left ventricular ejection fraction; HR: hazard ratio; VE/VCO2 at AT: ventilatory equivalent for carbon dioxide at anaerobic threshold.
